# Cerebral macro- and microcirculatory blood flow dynamics in successfully treated chronic hypertensive patients with and without white mater lesions

**DOI:** 10.1038/s41598-020-66317-x

**Published:** 2020-06-08

**Authors:** Martin Müller, Mareike Österreich, Lehel Lakatos, Alexander von Hessling

**Affiliations:** 1 Department of Neurology, Lucerne Kantonsspital, Spitalstrasse, CH-6000 Lucerne Switzerland; 2 Department of Radiology, Section Neuroradiology, Lucerne Kantonsspital, Spitalstrasse, CH-6000 Lucerne Switzerland

**Keywords:** Physiology, Diseases, Medical research, Neurology, Pathogenesis, Risk factors

## Abstract

The mechanisms of high blood pressure (HBP) -related brain pathology progression remain relatively unclear. We investigated whether lowering BP in chronic HBP patients normalizes cerebral perfusion dynamics at resistance vessel and capillary levels. Sixty-seven patients with HBP and 49 age- and sex-matched healthy controls underwent simultaneous recordings of middle cerebral artery blood flow velocity (CBFV), BP, and end-tidal CO_2_ concentration. Thirty-four controls and 28 patients underwent additional near-infrared spectroscopy recordings (oxygenated [O_2_Hb] and deoxygenated [HHb] hemoglobin). Degree of microcirculatory white matter lesions was graded by Fazekas scale. Dynamic cerebral autoregulation (dCA) was assessed by transfer function analysis. BP was successfully lowered (patients = 89 ± 15 mm Hg, controls = 87 ± 17), but cerebrovascular resistance was higher in BP patients (p < 0.05). BP-CBFV phase was lower in very low frequency (VLF) (left/right: 48 ± 20°/44 ± 17; controls: 61 ± 20/60 ± 21; p < 0.001) and low frequency (LF) (34 ± 14/35 ± 14; controls: 48 ± 20/44 ± 17; p < 0.05) ranges. Gain was higher in VLF range (in %/ mm Hg 0.56 ± 0.44/0.59 ± 0.49; controls: 0.32 ± 0.29/0.34 ± 0.32; p ≤ 0.005). BP-CBFV phase and gain did not differ across Fazekas groups. Across all patients, the capillary phases and gains (CBFV-[O2Hb], CBFV-[HHb]) were comparable to controls. Successfully treated chronic HBP results in normal brain capillary hemodynamics while the resistance vessel state is disturbed (phase decrease, gain increase).

## Introduction

The cerebral consequences of chronic high blood pressure (HBP) are manifold: the most frequent are hemorrhagic and nonhemorrhagic stroke, cerebral small vessel disease with white matter lesions (WML), microbleeds, or brain atrophy which can lead to cognitive decline and dementia^1^. Small vessel disease has also been reported to contribute to the pathogenesis of Alzheimer’s disease^2,3^. The pathological mechanisms of HBP-related brain pathology progression, however, are unclear. Among others, a constant elevated level of mean systolic or diastolic BP and their variabilities^4,5^, autonomic BP regulation disturbances^6^, increased pulse pressure amplitudes^7^, ischemic effects on the brain due to cerebral autoregulation (CA) failure^8–11^, and capillary dysfunction^12^ are proposed mechanisms of continuous cerebral tissue damage. More recently, aging is assumed to accelerate the HBP-driven pathologies^3,13^.

Cerebral blood flow is determined by BP-dependent regulatory effects and by metabolic influences on the resistance vessels via feedback mechanisms. These regulatory mechanisms can be observed noninvasively by cerebral blood flow velocity (CBFV) and its wave form, and by changes in the microcirculatory concentrations of oxygenated and deoxygenated hemoglobin (Hb) via near-infrared spectroscopy (NIRS)^14–16^. Anatomically, CBFV mirrors the regulatory effects in front of the resistance vessel in the large (macrocirculatory) brain supplying arteries such as the middle cerebral artery, while the concentration changes in oxygenated and deoxygenated Hb indicate effects behind the resistance vessel predominantly in the capillary bed. For clarification, the resistance vessels are part of the cerebral microvasculature and cannot directly be assessed noninvasively but only deducibly in the upstream macrocirculatory vessels. The other part of cerebral microvasvulature is the capillary bed which is approachable via NIRS. Together with noninvasive estimation of BP, CBFV and NIRS parameters offer sufficient resolution to analyze the dynamics of the regulatory processes from BP to the venous capillary bed.

A frequently-used approach to describe these dynamics is the estimation of phase shift and gain derived from transfer function analysis (TFA)^15–21^: at a given cycle of BP and CBFV changes, e.g. BP and CBF changes with a cycle duration of 10 seconds (=0.1 Hz) gain describes the power transformation from BP to CBFV, and phase shift indicates how much earlier or later in time the BP cycle will be found in the CBFV. A characteristic finding is that BP cycles around 0.1 Hz are delayed by approximately 1.10–1.70 seconds compared to the corresponding CBFV cycle. In patients with recently diagnosed, untreated HBP, or in subjects in which BP was elevated by phenylephrine, gain was shown to be reduced and the phase was either unchanged or decreased^22–24^; after lowering BP in these two populations, the phase shift and gain normalized. However, this reversibility might not be present in patients with chronic HBP^25^. We used this dynamic approach in accordingly treated chronic HBP patients and hypothesize that the regulatory effects of the resistance vessels as indicated by phase and gain are normalized in the macrocirculatory system BP - CBFV (assessed in the middle cerebral artery). Consequently, if treatment normalizes these resistance vessel measures, the capillary blood flow compartment should then be responsible for small vessel disease propagation. Our second hypothesis, therefore, is that the TFA parameters and/or the capillary transit time between CBFV and the concentrations of oxygenated Hb or deoxygenated Hb demonstrate pathological results which ideally should additionally demonstrate a relationship with the amount of WMLs as classified by the Fazekes scale^26^ score.

## Material and Methods

### Study sample and design

The study was approved by the Ethics Committee of Northwest and Central Switzerland and was performed in accordance with the Declaration of Helsinki, using good standards of clinical practice. Written informed consent was obtained from all participants. All data is available on request from the authors.

The control population consisted of 49 healthy individuals (females 15/males 34, mean age ± standard deviation [SD], 62 ± 11 years, range 42–87 years) (Table 1). Bilateral transcranial Doppler ultrasound recordings were not possible for all controls, so we report left (n = 49) and right (n = 47) middle cerebral arteries separately. All controls were free of known diseases and were non-smokers. The controls were recruited from hospital staff members after advertising the study among them.

**Table 1:** Comparison of baseline characteristics between control and the patient group.

	Controls (n = 49)	Patients (n = 67)	T-Test
Age (years)	62 ± 11	65 ± 13	ns
Female/male	15/34	18/49	ns
Mean blood pressure (mm Hg)	87 ± 17	89 ± 15	ns
Systolic blood pressure (mmHg)	115 ± 16	118 ± 21	ns
Diastolic blood pressure (mmHg)	64 ± 16	63 ± 17	ns
Mean Blood pressure variability (mm Hg)	L, 17 ± 4 R, 16 ± 3	L, 19 ± 5 R, 19 ± 5	L, df 115, t = −2.38, p < 0.05,d = 0.44 R, df 113, t = −2.88, p < 0.005, d = 0.72
Heart rate (beats/min)	68 ± 9	lL, 66 ± 10 R, 66 ± 11	ns ns
ETCO_2_ (mm Hg)	40 ± 3.9	39 ± 5.0	ns
Cerebral blood flow velocity (cm/s)	L, 45 ± 10 R, 57 ± 13	L, 53 ± 13 R, 52 ± 12	L, ns R, ns
Cerebrovascular resistance (mm Hg / cm/s)	L, 1.43 ± 0.43 R, 1.46 ± 0.41	L, 1.75 ± 0.52 R, 1.77 ± 0.44	L, df 115, t = −1.95, p = 0.05, d = 0.67 R, df 113, t = −3.66, p < 0.005, d = 0.72
Antihypertensive Drug class used			
Ca-Antagonists		23	
ACE inhibitors		29	
AT1 receptor inhibitors		16	
Diuretics		18	
β-Blockers		12	
Patient therapy with drugs from		1 class 32 2 classes 12 3 classes 10 4 classes 3	

ETCO2: end-tidal carbon dioxide concentration.

The patient group consisted of 67 consecutive HBP patients (females 18/males 49; mean age 65 ± 13, range 39–88 years, Table 1), who were referred to our neurovascular facilities for diagnosis of cerebrovascular diseases and met the study inclusion criteria (see below). Apart from dedicated stroke syndroms other reasons for referral were, e.g., unsystematic dizziness, the patient’s wish to estimate his/her general stroke risk, or unclassified visual disturbances with normal ophthalmologists findings. Our routine work-up of such patients consists of 2 stages: on stage 1 all necessary investigations are made to reach a definite diagnosis; stage 2 is a clinical re-evaluation after 3 months focusing on the controlling of the vascular risk factors. At the end of stage 1 eligible patients were then asked to participate in the study. We considered this time point the time point of study enrollment. To generate a homogeneous group of HBP patients, patients were eligible for the study when they met the inclusion criteria: known history of essential (not secondary) HBP (systolic BP ≥ 140 mmHg or diastolic BP ≥ 90 mmHg) for ≥2 years, a complete medical history and medical examination, presence of a cranial magnetic resonance imaging (MRI; Philips Achiva 3 Tesla [Philips, Netherlands] device or Siemens Magnetom 3T [Siemens, Germany], using DWI-, T2- and Flair-sequences]), presence of a duplex ultrasound examination of the brain supplying extra- and intracranial arteries (Acuson Antares S2000, Siemens, Germany), whereby ≥50% stenosis or occlusion of the large extra- or intracranial arteries was excluded, absence of other (than HBP) vascular risk factors such as smoking, diabetes mellitus, obesity (body mass index >30 kg/m^2^), absence of cardiac arrhythmias (atrial fibrillation), or a clinically manifest heart failure, no new symptoms suggestive for a ischemia relapse. Only treated dyslipidemia was allowed as an additional risk factor. After enrollment BP was controlled by regular follow-up visits to general physicians for 3 months. The study investigations were performed after these 3 months time periods at stage 2. Upon enrollment, the final diagnoses were microangiopathic minor stroke in 18 patients, transient ischemic attack in 12, and 37 patients were asymptomatic. All MRIs were examined by the same neuroradiologist (AvH), who made assessments about the presence of WML according to the grading schema by Fazekas *et al*.^26^: WML originate from microvascular changes, and the amount of WML correlate with the severity of small vessel disease (grade 1 slight, 2 = moderate, 3 = severe WML/small vessel disease); the Fazekas grading schema is robust, exhibits a good inter-rater reliability when grading is based on MRIs, and is clinically widely used. We graded MRIs without any WML as Fazekas 0.

### Experimental setting and instrumentation

All investigations were performed in the late morning with subjects in a supine position with approximately 30° head elevation in a dimly lit room. Coffee or tea was last ingested a ≥4 hours before to the beginning of the investigation.

As described in detail in a previous report^18^, we assessed

- The capillary blood flow by recording the concentration changes in oxygenated and deoxygenated Hb over time using the NIRO-200NX NIRS device (Hamamatsu Photonics, Herrsching, Germany). Infrared light is emitted at 3 frequencies (735, 810, 850 nm) through the skull and absorpt in the upper cortical layers by oxygenated Hb and deoxygenated Hb. Both Hbs backscatter the light with different intensities which correlate with the concentrations of oxygenated and deoxygenated Hb. We used self-adhesive NIRS probes: the detecting probe was placed at the lateral frontotemporal skull to ensure it was above the MCA territory, and the emitting probe was placed at a fixed distance of 4 cm at the frontal skull. After initial adjustments to determine the baseline hemoglobin concentration (in µmol/L), the NIRS device provided continuous percentage changes in oxygenated and deoxygenated Hb concentrations.
- The cerebral blood flow velocity in the middle cerebral artery using transcranial Doppler (TCD) ultrasound (MultidopX, DWL; Compumedics, Germany, 2 MHz probes). After attaching the NIRS probes, a head holder to affix the TCD probes was positioned and both middle cerebral arteries were identified using common criteria (CBFV directed towards the probe; depth of insonation 45–60 mm). End-tidal CO_2_ was measured via a small nostril tube connected to a capnograph embedded in the TCD device.
- Blood pressure by finger plethysmography (Finometer Pro; Finapres Medical Systems, Netherlands) placed at the fingertip of the right index finger with special attention to calibrate it to the brachial artery. The blood pressure signal was imported into the TCD device and simultaneously recorded along with the cerebral blood flow velocity and End-tidal CO_2_.

After all probes were placed and participants were familiar with their surroundings and the experimental setting, we began recordings of ≥5 min.

### Data preparation and analysis

Cerebral blood flow velocity, blood pressure, and End-tidal CO_2_ data were collected at 100 Hz. Oxygenated and deoxygenated Hb concentration changes via NIRS were collected at 5 Hz. The data were analyzed using Matlab (2018b; Math Works Inc., Natick, MA, USA). Only artifact-free 5 min periods were used. After aligning the common starting point, each raw data time series was resampled by averaging over 1 s intervals (one second = 100 data points for cerebral blood flow velocity, blood pressure, and End-tidal CO_2_, and 5 data points for oxygenated/deoxygenated Hb, respectively). The mean of the data series was then subtracted in each data series. Form these resampled and mean subtracted time series, the coherence and the TFA estimates of phase (in degrees) and gain between the different time series were extracted from their respective power auto-spectra or cross-spectra using Welch’s averaged periodogram method with a Hanning window length of 100 s, a window overlap of 50%, and a total Fast Fourier Transformation data length of 300 s, thereby allowing for calculations over a frequency range of 0.02–0.40 Hz.

Blood pressure - cerebral blood flow velocity TFA results are reported over the total frequency range (very low frequency range [VLF]: 0.02–0.07 Hz; low frequency range [LF]: 0.07–0.15 Hz; high frequency range [HF]: 0.15–0.40 Hz). Because coherence has been shown to be low in frequency ranges >0.15 Hz when oxygenated and deoxygenated Hb concentrations are involved^18^, we decided to use the frequency range of 0.02–0.15 Hz only for analysis which included NIRS parameters. To calculate means over each frequency range, we averaged phase and gain values with a coherence ≥0.34^17^. Cerebral mean transit times were calculated as phase differences between phase (cerebral blood flow velocity –[deoxygenated Hb]) – phase (cerebral blood flow velocity - [deoxygenated Hb]). In addition, cerebrovascular resistance was calculated as mean blood pressure /mean cerebral blood flow velocity, and heart rate was derived from the blood pressure signal trace.

### Statistical analysis

Matlab Statistical Toolbox was used for all data analyses. Using a Kolmogorov-Smirnov test, all data were shown to have a normal distribution, and the data are reported as mean ± SD. We used the t-test for comparisons between the 2 participant groups, and one-way analysis of variance (ANOVA), which includes a correction for multiple comparisons (Tukey-Kramer procedure) for within-group comparisons. A regression analysis was used to test whether the results showed any dependency on End-tidal CO_2_, BP variability (as calculated by the SD of mean BP), cerebrovascular resistance, or age. If 2 or more variables predicted a target variable, stepwise regression analysis was used to determine their effects. P-values ≤ 0.05 were considered statistically significant.

## Results

Patient and control groups were not different regarding age, sex, mean BP levels, End-tidal CO_2_, and heart rate. BP variability and cerebrovascular resistance were significantly larger in the HBP patients than in controls (Table 1). In the controls, increasing age was associated only with increasing cerebrovascular resistance (left: F(1,48) = 9.07, R^2^ = 0.162, t = 3.01, p < 0.01; right: F(1,46) = 8.20, R^2^ = 0.148, t = 2.86, p < 0.01) and decreasing CBFV (left: F(1,48) = 7.90, R^2^ = 0.144, t = −2.81, p < 0.01; right: F(1,46) = 11.5, R^2^ = 0.196, t = −3.38, p < 0.01); all TFA parameters were unrelated to age. In the patient group, neither cerebrovascular resistance, nor CBFV, nor TFA parameters were age-related.

**Figure 1:**
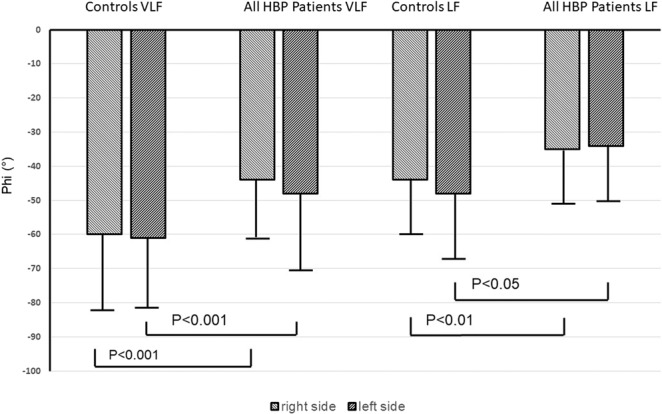
Phase shift (Phi) between controls and all high blood pressure (HBP) patients in the very low (VLF) and low frequency (LF) range. Statistical analysis performed by t-test. Error bars indicate standard deviation.

**Figure 2:**
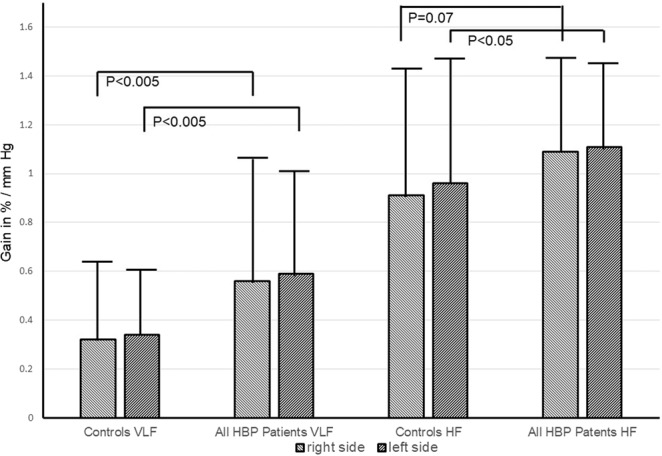
Comparison of gain (in %CBFV change/ mm Hg) between controls and all high blood pressure (HBP) patients in the very low frequency (VLF) and high frequency (HF) ranges. Statistical analysis performed by t-test. Error bars indicate standard deviation.

In the HBP patients, 66 left and 65 right middle cerebral arteries could be assessed by transcranial Doppler ultrasound. In TFA of the BP-CBFV systems (Table 2), the phase was significantly decreased (mathematically, however, becoming less negative) in patients from the VLF (0.02–0.07 Hz) and LF (0.07–0.15 Hz) ranges (Fig. 1). Gain (measured in cm/s/mm Hg and in %/ mm Hg) was significantly increased in the VLF range (Fig. 2); %/ mmHg gain was increased by trend in the HF range. Using ANOVA within the patient group (Table 3), there were no significant differences between the Fazekas groups regarding phase (all frequency ranges), gain (all frequency ranges), cerebrovascular resistance, and BP variability (both sides). In univariate regression models, increasing Fazekas group severity was not associated with any of these variables except age (left: F(1,66)=24.0, R^2^ = 0.24, t = 4.09, p < 0.005, d = 0.93; right: F(1,64) = 22.6, R^2^ = 0.26, t = 4.75, p < 0.005, d = 0.90). Regarding the initial clinical presentation, there were no differences in TFA parameters between asymptomatic patients, formerly symptomatic patients with transient ischemic attack, or those with minor stroke.

**Table 2:** Transfer function estimates in the macrocirculatory blood pressure – middle cerebral artery blood flow velocity system in controls and patients.

	Controls (n = 49)	Patients Left: n = 66; right: n = 65)	T-Test
Coherence			
-VLF	L, 0.51 ± 0.15 R, 0.49 ± 0.15	L, 0.54 ± 0.17 R, 0.56 ± 0.16	ns ns
-LF	L, 0.72 ± 0.14 R, 0.71 ± 0.13	L, 0.68 ± 0.17 R, 0.50 ± 0.17	ns ns
-HF	L, 0.62 ± 0.15 R, 0.65 ± 0.13	L, 0.62 ± 0.15 R, 0.65 ± 0.13	ns ns
Gain (cm/s / mm Hg)			
-VLF	L, 0.24 ± 0.22 R, 0.21 ± 0.25	L, 0.33 ± 0.27 R, 0.32 ± 0.29	df 115, t = −1.94, p = 0.05, d = 0.36 df 113, t = −2.23; p < 0.05, d = 0.40
-LF	L, 0.52 ± 0.25 R, 0.52 ± 0.22	L, 0.50 ± 0.23 R, 0.49 ± 0.21	ns ns
-HF	L, 0.58 ± 0.31 R, 0.59 ± 0.35	L, 0.59 ± 0.23 R, 0.59 ± 0.25	ns ns
Gain (%/mm Hg)			
-VLF	L, 0.32 ± 0.29 R, 0.34 ± 0.32	L, 0.56 ± 0.44 R, 0.59 ± 0.49	df 115, t = −3.156, p < 0.005, d = 0.64 df 113, t = −2.99, p < 0.005, d = 0.60
-LF	L, 0.84 ± 0.46 R, 0.86 ± 0.38	L, 0.89 ± 0.37 R, 0.87 ± 0.34	ns ns
-HF	L, 0.91 ± 0.50 R, 0.96 ± 0.51	L, 1.09 ± 0.40 R, 1.11 ± 0.36	df 115,t = −2.161, p < 0.05, d = 0.36 df 113, t = −1.801, p = 0.07, d = 0.29
Phase (°)			
-VLF	L, −61 ± 20 R,−60 ± 21	L, −48 ± 20 R, −44 ± 17	df 115, t = 3.696, p < 0.001, d = 0.65 df=113, t = 4.290, p < 0.001, d = 0.83
-LF	L, −48 ± 20 R, −44 ± 17	L, −34 ± 14 R, −35 ± 14	df 115, t = 2.769, p < 0.01, d = 0.81 df 113, t = 2.013, p < 0.05, d = 0.57
-HF	L, −16 ± 22 R, −10 ± 20	L, −13 ± 13 R, −13 ± 13	ns ns

VLF: very low frequency; LF: low frequency; HF: high frequency; L: left; R: right.

**Table 3:** Comparison of baseline characteristics and the transfer function estimates in the blood pressure-cerebral blood flow velocity system stratified according to Fazekas grade.

	Fazekas group 0		1		2		3		ANOVA	
	Left (n = 27)	Right (n = 27)	Left (n = 16)	Right (n = 15)	Left (n = 12)	Right (n = 11)	Left (n = 12)	Right (n = 12)	Left	Right
Age (years)	58 ± 11	58 ± 11	65 ± 11	65 ± 11	70 ± 15	69 ± 15	76 ± 8	76 ± 8	F(3,65)=7.88 p < 0.005, d = 1.38	F(3,63)=7.33 p < 0.005, f = 0.57 d = 1.38
									significant for	
									0 vs 2,3 1 vs 3	0 vs 2,3 1 vs 3
Mean BP (mm Hg)	95 ± 15	95 ± 15	84 ± 13	85 ± 13	92 ± 15	92 ± 16	87 ± 12	86 ± 12	ns	ns
BP variability (mmHg)	18 ± 5	18 ± 5	19 ± 4	19 ± 4	22 ± 7	21 ± 7	18 ± 4	18 ± 4	ns	ns
Heart rate (beats/min)	64 ± 10	64 ± 10	65 ± 11	66 ± 10	66 ± 9	66 ± 10	67 ± 11	67 ± 11	ns	ns
ETCO_2_ (mm Hg)	40 ± 5	40 ± 5	38 ± 5	38 ± 5	39 ± 5	40 ± 6	38 ± 5	38 ± 5	ns	ns
CVR (mm Hg/cm/s)	1.8 ± 0.5	1.8 ± 0.5	1.6 ± 0.4	1.7 ± 0.4	1.9 ± 0.7	1.8 ± 0.5	1.7 ± 0.5	1.8 ± 0.5	ns	ns
Gain (cm/s / mm Hg)										
-VLF	0.38 ± 0.34	0.41 ± 0.35	0.31 ± 0.20	0.27 ± 0.22	0.32 ± 0.27	0.26 ± 0.21	0.23 ± 0.19	0.24 ± 0.24	ns	ns
-LF	0.53 ± 0.22	0.48 ± 0.17	0.52 ± 0.17	0.52 ± 0.28	0.43 ± 0.30	0.43 ± 0.27	0.47 ± 0.27	0.51 ± 0.26	ns	ns
-HF	0.61 ± 0.26	0.59 ± 0.23	0.61 ± 0.17	0.64 ± 0.32	0.50 ± 0.22	0.54 ± 0.20	0.61 ± 0.27	0.59 ± 0.28	ns	ns
Gain (% / mmHg)										
-VLF	0.70 ± 0.56	0.75 ± 0.59	0.51 ± 0.36	0.50 ± 0.49	0.42 ± 0.29	0.45 ± 0.33	0.43 ± 0.30	0.48 ± 0.35	ns	ns
-LF	0.97 ± 0.33	0.88 ± 0.29	1.01 ± 0.28	0.91 ± 0.36	0.59 ± 0.41	0.78 ± 0.35	0.87 ± 0.44	0.95 ± 0.37	ns	ns
-HF	1.13 ± 0.36	1.12 ± 0.31	1.22 ± 0.36	1.20 ± 0.39	0.88 ± 0.42	1.01 ± 0.34	1.15 ± 0.42	1.18 ± 0.43	ns	ns
Phase (°)										
-VLF	−48 ± 17	−48 ± 12	−48 ± 25	−39 ± 18	−46 ± 22	−44 ± 26	−53 ± 21	−48 ± 16	ns	ns
-LF	−33 ± 13	−35 ± 15	−34 ± 14	−32 ± 16	−39 ± 18	−36 ± 13	−34 ± 11	−36 ± 12	ns	ns
-HF	−15 ± 15	−15 ± 15	−17 ± 12	−11 ± 15	−12 ± 11	−15 ± 10	−7 ± 10	− 7 ± 11	ns	ns

BP: Blood pressure; ETCO2: end-tidal carbon dioxide concentration; CVR: cerebrovascular resistance; VLF: very low frequency; LF: low frequency; HF: high frequency.

We used the CBFV-NIRS parameter to characterize the capillary dynamics. For all patients, concentration changes in oxygenated Hb followed CBFV about 50°; the concentration changes in deoxygenated Hb followed about 260–280° without many differences between VLF and LF ranges. These phase values did not differ from the corresponding values in controls (Table 4). %/% gain (measured as %NIRS parameter change / %CBFV change) also did not differ between patients and controls in both frequency ranges. Mean transit times in the LF range were calculated at 0.1 Hz (for comparison with the literature^15^) also did not differ between controls and the patient group (range: 4.14–4.84 s).

**Table 4:** Comparison of microcirculatory transfer function estimates between controls and patients.

	Controls (n = 34)	Patients (n = 28)	T-Test P
CBFV-[O_2_Hb]			
Coherence			
-VLF	L, 0.48 ± 0.08 R, 0.48 ± 0.10	L, 0.47 ± 0.10 R, 0.54 ± 0.13	ns ns
-LF	L, 0.51 ± 0.11 R, 0.54 ± 0.13	L, 0.53 ± 0.12 R, 0.51 ± 0.11	ns ns
Gain (%/%)			
-VLF	L, 0.46 ± 0.37 R, 0.48 ± 0.35	L, 0.40 ± 0.29 R, 0.53 ± 0.25	ns ns
-LF	L, 0.29 ± 0.18 R, 0.33 ± 0.22	L, 0.33 ± 0.20 R, 0.40 ± 0.37	ns ns
Phase (°)			
-VLF	L, 52 ± 37 R, 43 ± 38	L, 54 ± 29 R, 45 ± 32	ns ns
-LF	L, 48 ± 20 R, 51 ± 33	L, 54 ± 29 R, 45 ± 32	ns ns
CBFV-[HHb]			
Coherence			
-VLF	L, 0.48 ± 0.09 R, 0.48 ± 0.10	L, 0.49 ± 0.19 R, 0.50 ± 0.13	ns ns
-LF	L, 0.47 ± 0.13 R, 0.49 ± 0.10	L, 0.48 ± 0.08 R, 0.48 ± 0.10	ns ns
Gain (%/%)			
-VLF	L, 0.21 ± 0.13 R, 0.18 ± 0.12	L, 0.22 ± 0.27 R, 0.22 ± 0.15	ns ns
-LF	L, 0.10 ± 0.09 R, 0.16 ± 0.19	L, 0.13 ± 0.14 R, 0.14 ± 0.10	ns ns
Phase (°)			
-VLF	L, 258 ± 44 R, 256 ± 44	L, 268 ± 49 R, 279 ± 45	ns ns
-LF	L, 284 ± 38 R, 259 ± 36	L, 279 ± 36 R, 280 ± 44	ns ns
Mean transit time (°)			
CBFV-[O_2_Hb] – CBFV-[HHb]			
-VLF	L, 204 ± 51 R, 214 ± 39	L, 214 ± 56 R, 242 ± 58	ns ns
-LF	L, 242 ± 46 (4.84 ± 0.92) R, 207 ± 51 (4.14 ± 0.11)	L, 223 ± 47 (4.46 ± 0.94 R, 223 ± 54 (4.46 ± 1.08)	ns ns

BP: blood pressure; [O_2_Hb]: concentration of oxygenated hemoglobin; [HHb]: concentration of deoxygenated hemoglobin; Coh: Coherence; CBFV: cerebral blood flow velocity; L: left; R: right. Mean transit time in phase (°) and, for comparison with the literature, in s after transforming to time domain at 0.1 Hz (in parenthesis). VLF: very low frequency; LF: low frequency.

## Discussion

Physiologically, the main purpose of the cerebral resistance vessel is to provide a relatively constant blood supply to the brain’s capillary bed. These efforts can be characterized by functional/dynamical parameters such as phase and gain, or by static CBF measurements via e.g. perfusion computed tomography or perfusion MRI technology^10,11^. High BP leads to changes in the dynamics (functional capacity) of the resistance vessel which can be derived from analyzing the (macrocirculatory) BP-CBFV relationship. Sufficient treatment of HBP should consecutively normalize any disturbances in the BP-CBFV relationsship. We, therefore, hypothesized that the propagation of small vessel disease could be allocated at the capillary level of cerebral blood flow regulation by demonstrating disturbed TFA parameters here. In contrast to our hypotheses however, we found that, despite normalizing BP, the cerebral blood flow regulating system is still disturbed at the resistance vessel level as indicated by the BP-CBFV system, and that the cerebral blood flow regulating system is normal at the capillary level indicating that the main task of the resistance vessel is still achieved.

The elevated cerebrovascular resistance in our patient group was not normalized and dynamic CA remained disturbed despite sufficient BP lowering. This suggests that the continuously-elevated cerebrovascular resistance trended toward (1) either an incomplete structural recovery of resistance vessels from vessel rarefication, (2) lumen narrowing, or (3) an ongoing and not necessarily pressure-dependent vessel dysfunction as a reason for the elevated resistance (e.g. endothelial dysfunction, humoral influences)^3,9,27–33^. If we consider structural vessel changes only, then our cohort represents patients at a disease stage with irreversible resistance vessel changes. In this condition, dynamic CA responded with gain increase and phase decrease. Similar findings were reported by Immink *et al*.^34^ in a small number of patients with long-term hypertension who also presented with secondary hypertensive ocular complications. Together, these findings may hint towards a suggestion that the dynamic CA response of patients at an advanced disease stage is distinctly different from that of healthy subjects or patients with recently diagnosed HBP; in these latter patients, gain decreases and normalizes after treatment, and phase changes are usually not present^22–24,35^. A low gain in an arterial vessel indicates a good ability to buffer (BP elevations, for example), and a high gain indicates a loss of this ability^36^. Thus, at an early HBP disease stage, the CA responds at the resistance vessel level with a gain decrease indicating an adequate capability to buffer BP increases; and at an advanced stage, CA responds with a phase decrease and a gain increase indicating a poor buffer capability leading to a high amount of energy to be transported transmurally. Speculatively, the increased BP variability in our patient group is also less well-buffered and could contribute to further brain damage.

The second result of our study was that, irrespective of the exact mechanism for the cerebrovascular resistance increase, the mechanisms of the cerebrovascular system consisted of compromised dynamics of CA in the BP-CBFV system, and normal dynamics of cerebral blood flow regulation at the capillary level. If HBP patients are sufficiently treated, our overall findings indicate that the extent of the hemodynamic compromise of the resistance vessels does not exceed a point at which the capillary compensatory mechanisms become effective, such as shortening of the mean transit time by opening up of functional shortcuts due to capillary transit time homogenization^9,12,37^ or of prolongation the mean transit time^15,38,39^ via delaying venous outflow^15^. Our results at the capillary level support recent CBF studies which demonstrate that cerebral hypoperfusion is not always present in patients with HBP^29,40,41^, and that an intensive BP lowering was tolerated well by patients with severe SVD^41^.

Neither the findings at the BP-CBFV level nor the findings at the capillary level demonstrated a correlation to the amount of WMLs. This contradicts our hypothesis that CBF regulation at the capillary level would be the more disturbed the lager the WML amount is. Our results are surprising and may favor the view that the primary location of small vessel disease propagation is at the resistance vessel level due to CBF regulation failure. However, Birns *et al*.^42^ used another method to test dynamic CA between BP and CBFV, and also found that dynamic CA disturbances did not correlate with the total amount of WML. Thus, BP-CBFV dynamic CA disturbances are quite similar across all Fazekas groups, making it difficult to attribute dCA at the resistance vessel level with the responsibility for the different SVD stages. Age and other factors (not investigated by us) may therefore be more relevant. Nevertheless, one has cautiously to keep in mind that our number of patients with severe WML was low, a fact that could have hindered the detection of relevant hemodynamic relationships.

## Limitations

In our patients CVR was still increased despite adequate BP lowering therapy. We assumed that this CVR increase is present in every patient irrespectively of its underlying condition such as an anatomical vessel rarefication, an increased vessel stiffness or an unknown metabolic change. Each of the used drug class influences the vascular tone/resistance differently^43^: Ca-Antagonist dilate the resistance vessel, ACE inhibitors and AT1- receptor blockers can vasodilate larger arteries but can also vasoconstrict small vessels; diuretics, ACE-inhibitors and AT1 receptor blockers lower BP additionally by reducing the intravascular volume. Thus, our results might be based on different pathophysiological conditions which met the different effects of each individual drug class. To this end, we cannot differentiate by our used methods which underlying condition meets which drug effect. In an additional multiple regression analysis with all drug classes as the independent variables in the model and phase and gain as the dependent (to be predicted) variable, the models did not reveal that any drug class predicted either gain nor phase. However, we consider the number of patients too low to allow for an analysis of such drug effects.

Given the penetration depth of NIRS wavelengths, it is assumed that the dynamic CA patency in the upper cortical layer was comparable to the deep white matter conditions. Whether this assumption is correct has not been demonstrated, as clinical experience suggests that the lesion load of WML in the deep white matter is higher than that in the subcortical brain tissue. Immink *et al*.^35^ (and the current study) used TFA on spontaneous CBF oscillation for analysis. Birns *et al*.^42^ used the thigh cuff test for dCA evaluation in their HBP patients, and also did not find any dependency of WML severity on dCA results. Thus, both analysis methods seemed comparable in this respect^42,44^ and might not fully portray the progression of HBP-related small vessel disease. We investigated roughly half of our patients 3 months after a clinical event, which may have influenced our results. Clinical events, especially strokes, can lead to a disturbed dynamic CA, although this is usually transient and resolves after 2 weeks^45,46^.

## Conclusions

In successfully treated patients with an HBP history of ≥2 years, BP lowering therapy results in an overall normal brain capillary hemodynamic state at the expense of the resistance vessel system that shows a disturbed dynamic autoregulation ability with phase decrease and gain increase. The increased gain indicates that the vessel’s ability to buffer pressure or flow across the vessel wall is compromised at CBF period lengths between 13 and 50 s. This dynamic approach to CA should be evaluated further to determine whether it could play a role in guiding HBP treatment in the future.
